# Barriers and enablers for practicing kangaroo mother care (KMC) in rural Sindh, Pakistan

**DOI:** 10.1371/journal.pone.0213225

**Published:** 2019-06-17

**Authors:** Qamar Zaman Jamali, Rashed Shah, Farhana Shahid, Aisha Fatima, Saraswati Khalsa, Jana Spacek, Presha Regmi

**Affiliations:** 1 Department of Health, Save the Children International, Islamabad, Pakistan; 2 Department of Global Health, Save the Children US, Washington DC, United States of America; 3 Technical Unit, Jhpiego, Karachi, Pakistan; 4 Jhpiego, Baltimore, United States of America; Liverpool School of Tropical Medicine, UNITED KINGDOM

## Abstract

**Background:**

More than 2.5 million newborns die each year, accounting for 47% of children dying worldwide before their age of five years. Complications of preterm birth are the leading cause of death among newborns. Pakistan is amongst the top ten countries with highest preterm birth rate per 1000 live births. Globally, Every Newborn Action Plan (ENAP) has emphasized on Kangaroo Mother Care (KMC) as an essential component of neonatal health initiatives.

**Materials and methods:**

We conducted this qualitative study with 12 in-depth interviews (IDIs) and 14 focus group discussion (FGD) sessions, in two health facilities of Sindh, Pakistan during October-December 2016, to understand the key barriers and enablers to a mother's ability to practice KMC and the feasibility of implementing and improving these practices.

**Results:**

The findings revealed that community stakeholders were generally aware of health issues especially related to maternal and neonatal health. Both the health care providers and managers were supportive of implementing KMC in their respective health facilities as well as for continuous use of KMC at household level. In order to initiate KMC at facility level, study respondents emphasized on ensuring availability of equipment, supplies, water-sanitation facility, modified patient ward (e.g., curtain, separate room) and quality of services as well as training of health providers as critical prerequisites. Also in order to continue practicing KMC at household level, engaging the community and establishing functional referral linkage between community and facilities were focused issues in facility and community level FGDs and IDIs.

**Conclusion:**

The study participants considered it feasible to initiate KMC practice at health facility and to continue practicing at home after returning from facility. Ensuring facility readiness to initiate KMC, improving capacity of health providers both at facility and community levels, coupled with focusing on community mobilization strategy, targeting specific audiences, may help policy makers and program planners to initiate KMC at health facility and keep KMC practice continued at household level.

## Introduction

More than 2.5 million newborns die each year, accounting for 47% of children dying before the age of five years worldwide [1]. Complications of preterm birth are the leading cause of death among newborns [2]. Of the fifteen million babies born too early each year, more than one million die due to complications related to preterm birth. Low birth weight (newborns weighing < 2,500 grams at birth), due to prematurity and/or restricted growth in utero, is also a major contributor of annual newborn and child deaths worldwide. Pakistan is amongst the ten countries with the greatest number of preterm births and the highest rates of preterm births per 1000 live births, with 860,000 babies born preterm and 75,000 children dying under the age of five due to direct preterm complications annually. Almost one in every three babies born in Pakistan (32%) is a low birth weight (LBW) baby [3]. World Health Organization (WHO) issued recommendations [4] for the care of preterm infants in November 2015, including kangaroo mother care (KMC). An international joint policy statement and endorsement also came from health professional associations (American Academy of Pediatrics, Council of International Neonatal Nurses, International Council of Nurses, American College of Obstetricians and Gynaecologists, International Federation of Gynaecology and Obstetrics, American College of Nurse-Midwives, International Paediatric Association and International Confederation of Midwives) for universal use of KMC for preterm and LBW Infants. The WHO has defined KMC as early, continuous, and prolonged skin–to–skin contact between the mother and babies; exclusive breastfeeding or breast milk feeding; early discharge after hospital–initiated KMC with continuation at home; and adequate support and follow–up for mothers at home[5]. KMC has three main components, including: thermal care through continuous skin-to-skin contact by being wrapped with a cloth to the bare chest of the care giver; support for exclusive breastfeeding or other appropriate feeding; and early recognition and response to complications [6]. Neonatal mortality analyses from a 2014 Cochrane review [7] (11 randomized controlled trials) and a 2016 meta-analysis by Boundy [8] (16 studies) found a 33 percent and 23 percent reduction in neonatal mortality, respectively, at the last follow-up when comparing KMC to conventional neonatal care. In both mortality analyses, all but two of the studies included were in resource poor countries. The systematic review conducted by Agudelo & Rosello [9] suggested KMC as an effective and safe alternative for conventional neonatal care for low birth weight (LBW), especially in settings with limited resources. In LBW newborns (< 2000 g) who are clinically stable, practicing KMC can reduce mortality and if widely applied could reduce deaths in preterm newborns [10].

Globally, after the Every Newborn Action Plan (ENAP) has identified KMC as an essential component of newborn health initiatives [11], many agencies have made KMC a priority. In Pakistan as well, policymakers, researchers and key stakeholders in newborn health have recently highlighted KMC as a top preterm intervention agenda as part of the ENAP [12].

United States Agency for International Development (USAID) led a successful initiative in Pakistan through provision of comprehensive maternal newborn child health (MNCH) services to women and children in Sindh Province. Maternal and Child Health Integrated Program (MCHIP) led by Jhpiego and in collaboration with Save the Children, Program for Appropriate Technologies in Health (PATH) and John Snow, Inc. (JSI) implemented this MNCH initiative across 16 districts during 2013–2018 in Sindh province. In addition, Save the Children was also implementing another health project with funding support from the Australian Government, titled Integrated Maternal, Newborn and Child Health (IMNCH) Sindh in Shikarpur and Jacobabad districts. The IMNCH-Sindh project was designed with the objective of complementing MCHIP interventions and activities in these two districts.

MCHIP/USAID and the Australian government supported IMNCH programs facilitated to introduce KMC in two districts (Khairpur and Shikarpur, respectively) in Sindh as a pilot study to introduce KMC first ever in Sindh, Pakistan, in order to make recommendations for scale-up the KMC intervention effectively in other districts of Sindh and to other provinces in the country. To achieve this, it was crucial to understand the key barriers and enablers contributing to the caregiver’s (in)ability to practice KMC and the feasibility of implementing and improving these practices.

In order to enhance successful initiation and implementation of KMC, study by Seidman et al [10] highlighted the importance of understanding the barriers and enablers, within the sociocultural and health systems context, those support uptake and continuity of KMC practice from both supply and demand side aspects.

This pre-implementation study aimed to identify the barriers and enablers prior to introducing KMC in Sindh province at both facility and community level in order to inform policy makers and program planners a way to roll out.

## Materials and methods

This study followed a qualitative study design and recruited study participants by employing purposive sampling strategy based on convenience and availability of eligible study participants.

### Study site and population

The study was conducted in two MCHIP and IMNCH supported hospitals in two districts–Khairpur and Shikarpur: 1) CEmONC hospital [Gambat Institute of Medical Sciences (GIMS)] in Khairpur district and 2) District Headquarter (DHQ) hospital in Shikarpur district. These selected health facilities were providing 24 hours continuous services for maternal, newborn and child health care.

According to the 2017 Census of Pakistan [13], districts Khairpur and Shikarpur had a population of 2.4 million and 1.2 million, respectively.

### Sampling frame and participants’ selection

A list of study participants was developed to include potential study participants to explore the factors both from demand and supply sides (i.e. at community and facility level, respectively). Each study hospital’s medical superintendent who is responsible for the administrative and management of the facility and district health officer (DHO) who is in charge of all health related activities at the district were selected to participate in the study to explore system level issues. Further all human resource engaged in paediatrics, obstetrics and gynaecology services [doctors, nurses, midwives, Lady Health Visitors (LHV)] in the study hospitals and lady health workers (LHWs) attached to the selected health facilities, working in the catchment population of health facility were approached to participate in the study. Based on their availability and consent status, data were collected from the health care providers (one from each category) at selected hospitals. For some positions (e.g., District health officer, Medical Superintendent, Paediatrician, Obstetrician/Gynaecologist) there was only one person posted in each study hospital. If that specific person was available, we included the person in the study upon taking consent. For couple of other positions (e.g., Midwife, Lady Health Visitor), several health care providers were posted; we included available and consenting ones from each study hospitals. From available LHWs who consented, we randomly selected 4–6 LHWs for one FGD per district.

From the registers of the study hospitals, we obtained names and addresses of recently delivered women (RDW) who gave birth to either a premature or full-term baby during three months prior to the study date. From this list, we randomly selected study participants and invited to participate in focus group discussions (FGDs). We invited only mothers whose babies were alive. In each district, we organized one separate FGD with each category of respondent (i.e., mothers who had preterm/LBW baby, mothers who had term baby, husband of RDWs, influential/decision making women in the household (senior female family members in the household like mother-in-law, sister-in-law). Size of each focus group varied from 4–6 participants. We contacted selected participants in advance and those who were available on the day of data collection and provided consent to participate in the FGD were engaged in the discussion. During these FGDs, the RDWs and senior female family members described their experiences about child birth, immediate newborn care. We assigned one moderator and one note taker for each FGD session. The moderator of the FGD briefly described the KMC method and showed them how to practice KMC using vignette. The participants were asked about their thoughts on benefits of practicing KMC, their ability to practice KMC, support or oppose from family, barriers those prevent practicing KMC, how community women could be encouraged to practice KMC. The moderator probed into participants’ discussion to gather detail and relevant information on each of these topics. Considering pivotal role of traditional birth attendants (TBAs) in conducting child delivery at home and immediate newborn care thereafter, we also selected available and consenting TBAs from same villages for FGD. Selected eligible respondents who were unable to attend the FGD session and those who didn’t provide informed consent were excluded from the study.

### Study procedure and data collection

We conducted one-to-one in-depth interviews (IDIs) and FGD sessions, in a place allowing convenience and privacy for the participants, to collect qualitative data. We developed respective field guidelines for each type of respondent by using open-ended questions and with potential probes to achieve study objectives. All data collection tools were translated from English into local Sindhi language and were also back translated to check quality of the translation. All interviews were conducted in local language.

Four trained interviewers and two supervisors were employed for conducting the IDIs and FGDs in the field. All team members were fluent in speaking and writing the local language (Sindhi); they had necessary skill and experience of collecting qualitative data.

All interviewers and supervisors were trained over five days on the study protocol, concept of KMC, basics on qualitative research methods, and how to effectively conduct IDIs and FGDs. The training included scenario-based practical exercises using research tools combined with theory-based lectures. Two teams were formed (each team had two interviewers and one supervisor); during FGD session, one interviewer performed the role of moderator while the other one acted as the note taker. In addition to taking notes, digital voice recorders were used with the consent from participants so that maximum information can be collected with little risk of data loss. A set of vignettes was also used to facilitate conducting the IDIs and FGDs. Data collection was continued until saturation occurred and no new theme or information arose.

### Data analysis procedures

Transcripts were transcribed verbatim, translated into English and personal identifiers were removed. Each translated transcript was checked for accuracy and quality. We followed the framework approach for analyzing qualitative data. A thematic framework was constructed from two main themes with subthemes using NVIVO software (version 9). The two main themes included supply and demand side barriers and enablers for implementation of KMC. Health service providers’ knowledge, skill and practices about newborn care, feasibility of initiation and continuation of KMC as well as needs for strengthening facilities to implement KMC intervention, and community linkages and referral system were the major subthemes on the supply side. On the demand side, sub themes were community knowledge and practices about birth and essential newborn care (ENC), specifically focused on cleanliness and thermal protection, immediate and exclusive breastfeeding, management of newborn illness and care of preterm and/or low birth weight (LBW) newborns; and acceptability of KMC at the household level.

Data were also compiled into charts while each chart was constructed under a thematic heading, the rows represented respondents (or cases) and the columns represented the subthemes. Charts were examined for each theme and each subtheme, which constituted a column in the chart, was read down across all cases. This allowed a better understanding of the range of data that existed within a subtheme and helped in understanding the different emerging categories and concepts. Perceptions of the respondents were explored in-depth by examining patterns in the data through comparing their accounts. A final thematic framework was derived at the final stage of data analysis and the study findings are presented alongside quotes from the interviews to present the perceptions of the respondents. Informed written consent was obtained from all individual study participants. This study received ethical approval from National Bioethics Committee, Pakistan (Ref. No: 4-87/16/NBC-216/RDC/1245; dated October 18, 2016). Also Ethical Review committee in Save the Children US approved to conduct this study.

### Ethical approval

This study received ethical approval from National Bioethics Committee, Pakistan (Ref. No: 4-87/16/NBC-216/RDC/1245; dated October 18, 2016). Also Ethical Review committee in Save the Children US approved to conduct this study.

## Results

A total of twelve IDIs and fourteen FGDs were conducted across both study sites. The numbers of participants in IDIs and FGDs in study districts are shown in Tables 1 and 2.

**Table 1:** Number of in-depth interviews (IDIs) conducted.

Study participants	# of IDIs in each district	Total # of IDIs in 2 districts
**District Health Officer**	1	2
**Medical Superintendent**	1	2
**Midwife**	1	2
**Lady Health Visitor**	1	1
**Paediatrician/ Neonatologist**	1	2
**Obstetrician/ Gynaecologist**	1	2
**Total IDIs**	6	12

**Table 2:** Number of focus group discussion (FGD) sessions conducted.

Participants in FGD sessions	# of FGD session in District Khairpur (# of participants)	# of FGD session in District Shikarpur (# of participants)	Total # of FGDs in 2 districts (# of participants)
**Lady Health Workers**	1 (5)	1 (6)	2 (11)
**Traditional Birth Attendants**	1 (4)	1 (4)	2 (8)
**Recently Delivered women (RDW) with LBW/premature birth**	1 (4)	1 (4)	2 (8)
**RDW with term live birth**	1 (5)	1 (5)	2 (10)
Influential/decision making women in the household (senior female family members^*^)	1 (5) 1 (6)	1 (6) 1 (4)	4 (21)
**FGD with Husband of recently delivered women**	1 (6)	1 (5)	2 (11)
**Total FGDs (# of participants)**	7 (35)	7 (34)	14 (69)

* For example: mother-in-law, sister-in-law, mother, sister, aunty. These senior female family members have influence on decision making, specifically for pregnant women and recently delivered women, on the issues regarding pregnancy care, child delivery care, care of newborn

We present our study findings in following two sections: A) Enablers and B) Barriers of KMC implementation.

### A) Enablers of KMC implementation

#### Support of health providers

The health managers and care providers at study hospitals were receptive and supportive of the idea to implement KMC in their respective health facilities and continuous practice of KMC at household level.

*“We will implement KMC because it is very cheap and no expensive equipment is required. So we will help to implement KMC at our facility” (Health care provider, IDI participant)*.

Health care providers believed that continuous practice of KMC at home can only be achieved if there is strong support from family as well as from community based outreach LHW/health facility staff to make sure KMC is practiced as recommended.

*“Someone should visit the mothers practicing KMC at home or they should come to hospital for regular follow up to make sure that they are practicing KMC correctly” (Paramedic staff, IDI participant)*.

#### Effective community linkage and referral system

The health care providers’ responses also revealed that in order to implement KMC at household level, it is important to engage the community, and there is need to create link between the household and health facilities to build the referral mechanism.

*“Strong community mobilization is required to initiate KMC at household level; this should target not only mother but also other members of family and community at large” (Gynaecologist, IDI participant)*.

The success of KMC implementation needs to engage LHWs in supporting mothers about appropriate use of KMC.

*“LHWs are the best option to implement practicing KMC at the household level because they regularly visit households in their catchment area” (Health care provider, IDI participant)*.

#### Awareness and concept of KMC at community level

Although community level participants (RDW and influencers on decision making in the family like mother-in-law) were found not aware previously about the concept of KMC, however, many of them were found to believe that low birth weight (LBW) and preterm newborns need more care than term newborns and may need hospitalization. These study participants were aware that preterm newborn need warmth hence covered them with more layers of clothing as compared to term newborns.

*“Low weight babies do not cry much and can’t feed properly because they are weak. They need more care (covering with more layers of cloth) than term babies (RDW, FGD participant)*.

*“Small and weak babies have less weight and they don’t feed at the time. They need more care, like follow up with the doctor (RDW, FGD participant)*.

#### Acceptance and support at household level

Supporting FGD data collected from community level study participants revealed that KMC will be acceptable and supported by family members and community because it improves health and survival of the preterm/LBW newborn. Furthermore, participants themselves pointed out the cost saving factor as other benefit of practicing KMC.

*“Yes*, *they (other family members*, *including husbands) will support us to do practice of KMC*. *It gives an opportunity to make baby healthy without paying any rupees” (RDW, FGD participant)*

### B) Barriers of KMC implementation

#### Lack of facility readiness to implement KMC

Managers and health care providers recommended facility strengthening as a prerequisite for implementing KMC at facility level. They pointed out that in-patient ward in the facilities need modifications to ensure privacy for mothers.

*“Health facilities need modifications in hospital wards for ensuring privacy to practice KMC and all necessary things required (to practice KMC) should be provided to the patient”(Health care provider, IDI participant)*.

#### Lack of training health workers

In addition to suggesting infrastructure modification at facility, the respondent managers and health service providers also talked about training need for facility staff. None of the health workers (care provider or manager), both at facility and community level, has ever received any training in KMC and they identified staff’s capacity strengthening as one of the basic requirements for implementing KMC at facility level.

*“Neither we (managers) nor our staff in this facility received any training on KMC ever. Training at all levels from management, providers and paramedic staff is a prerequisite before introducing KMC at facility” (Health manager, IDI participant)*.

*“First of all we (health care providers) should be trained in conducting KMC and counseling skills to convince mothers to practice” (Health care provider, IDI participant)*.

In addition to health care facility staff, community based outreach workers (e.g., LHWs) were also identified with lack of knowledge and experience about KMC, though few responses from few LHWs reflected their awareness on need of special and more care for preterm and LBW babies.

*“Neither I have ever seen this practice anywhere nor (I have) received any training on this before” (LHW, FGD participant)*.

#### Lack of time / workload of LHWs

With reference to involvement of LHWs in implementation of KMC, a concern was also raised due to their existing workload.

*“LHW can play very important role in implementing KMC hence (they) should be trained on KMC. There must be transport facility and other incentives for LHWs…… Having a trustworthy LHW would be the positive factor to encourage KMC at house level” (LHV, IDI participant)*.

*“LHWs are already overburdened in other priority health issues such as polio eradication*. *Hence their commitment is necessary to support mothers and referral newborn to facility in case of complication” (Health care provider-IDI participant)*

#### Socio-cultural norms and practice

Study participants also expressed concerns that other female family members may oppose practicing KMC since it will result in shifting of KMC practicing mother’s workload to other female member(s) in the family. It will be challenging for RDW too, who otherwise is expected to contribute to household chores after delivery, particularly if there is no other female in the family to support in the household.

*“Our senior family member may not allow me to do KMC at home” (RDW*, *FGD participant)*

*“I am only single living with husband in my home, so. I do my all work as sweeping, cleaning etc. at home” (RDW, FGD participant)*.

Additionally, study respondents raised few other socio-cultural concerns regarding acceptance and support to KMC at household. They were also afraid that they might also face ridicule if mothers spend most of time in caring of their newborns. Culturally they are expected to take care of household chores along with taking care of their newborns.

*“I have few concerns …it (practicing KMC) will affect (undertaking) home responsibilities of the household*, *face taunts of other family members that all the times she held baby with herself”(TBA, FGD participant)*

Some RDW participants raised concern regarding potential opposition to the practice of KMC due to prevalent social norms of privacy, given that practicing KMC needs the mother to put the baby on her bare chest, as appeared in the quote below:

*“In our culture women are not even permitted to go relatives’ home so, how a woman can practice KMC with half naked dress pattern”? LHW*, *FGD participants*

*“Grandfather will not allow practicing KMC because it is against social norms to wear such type of clothes which are designed for KMC” (TBA FGD participant)*.

The summarized results yielded from facility and community level respondents are listed below, in Table 3.

**Table 3:** Summarized results from respondents at health facilities and community.

Highlighted features from IDIs and facility level FGDs	Highlighted features from community level FGDs
• Training of health care providers • Separate rooms/curtains in wards to ensure privacy • Equipment and supplies needed to ensure functional unit for critical neonatal care • Water and Sanitation facilities to maintain hygiene • Supplies (e.g., KMC binder and clothes) for mother and newborn to practice KMC	• Community members were found aware that– - newborns should be protected against the cold; and - skin-to-skin could be a beneficial way to protect these babies at risk • But skin-to-skin contact to provide warmth is not the prevailing norm. • Support from family members is very crucial to continue KMC practice; specifically, support from mother-in-law and sister-in-law is crucial for the recently delivered mother to practice KMC. • Given the extended family structure in Pakistan, it is highly likely that other female family members will be able to support the mother to practice KMC. • Husbands are usually not the caregivers of the newborns and less likely to have any active role in practicing KMC. Although, husbands showed willingness to practice KMC for the health and survival of their newborns. Nonetheless, husbands may have little time to practice given their responsibilities of working outside home or they also may suffer challenges of prevailing customs and socio-cultural norm. • Mother’s obligation to perform household chores is one critical barrier to KMC practice and many mothers found KMC as a burden due to responsibilities at home or work

## Discussion

Our findings are consistent with results from past research on health worker and community members’ perspectives on the feasibility of introducing KMC as well as key barriers and enablers affecting implementation. Health care providers, both at facility and community level, received several newborn care trainings such as Helping Babies to Breathe (HBB), Essential Newborn Care (ENC), neonatal umbilical cord care by using chlorhexidine, and similar other relevant training courses. However, we found that none of the health providers and managers had received any previous training on KMC in the selected study facilities. The respondents at facility level emphasized the critical need for KMC training as a prerequisite of implementing KMC at facility level. Relevant evidence exists in literature and echoes our findings as training opportunities are crucial to provide health care workers with knowledge and skills to facilitate KMC [14]. In a multi country study from Malawi, Mali, Rwanda, and Uganda, researchers found that in service training and including KMC in preservice curricula is a facilitator to implement KMC [15]. Expanding training to other health-care personnel, such as administrators and interns, also enabled KMC acceptance. Another study result also revealed in-service training of health-care workers as enhancer of KMC implementation [16]. Many nurses also found that integration of KMC into pre-service and training curricula was beneficial to accelerate its uptake [17, 18].

Our study results also revealed health care managers and providers at study hospitals are supportive of the idea of implementing KMC, which is a crucial enhancer, and offers opportunity to initiate KMC at facility level and rollout to community level. To establish an effective KMC at the facility level, motivation and enthusiasm of health care provider towards KMC is essential. Evidence in the literature from previously conducted studies are found supporting this finding. The above mentioned multi-country study in Africa on implementing facility-based kangaroo mother care services also found the staff as enthusiastic about KMC services and the support of senior management at the district and facility level played an important role in upholding staff motivation and their ability to continue KMC service [15]. Often, nurses want to practice KMC at their facility, but a lack of managerial support and poor staffing levels impede its practice [19]. Facilities that had successfully implemented kangaroo mother care reported that they received support from their management and had good communication among the staff [20–22].

In a systematic review, researchers identified "issues with facility environment/resources" and "negative impressions of staff attitudes or interactions" as the top two barriers to practice KMC at the facility setting [10].

Strengthening health facilities is an essential prerequisite for introducing KMC implementation at the facility level, as revealed from our study results. This includes infrastructure, equipment and other required logistics and materials for KMC at the facility. Several studies also identified that inadequate resources and logistics (e.g., chairs, beds, linens, curtains, KMC wraps, etc.), insufficient physical space and inadequate privacy (e.g., lacking private space or privacy screen) are major hindering factors to support KMC at the facility level [22–25]. Previous studies have found that when there were adequate logistics (beds, chairs, KMC wrap/binder), as well as physical space with adequate privacy where mothers could practice KMC, the adoption and uptake of KMC were easier at those facilities [22, 26, 27].

Our study respondents emphasized on the need of community level awareness raising and creating links between community and health facilities through LHWs for better implementation at facility as well as continuation of KMC practice at household. The role of LHWs is key factor in improving acceptance and compliance of KMC at facility and community level, respectively. Effective counseling of mothers and their household members is the key for acceptance and successful adherence to the protocol of KMC. As our study respondents suggested, these counseling and awareness raising sessions for community could be facilitated using different media such as video sessions and other information, education and counseling (IEC) materials during household visits by LHWs. At facility level, nursing staff is mainly involved in counseling and at the community level, LHWs are the key player to create awareness and ensure acceptance and adherence. Evidence also supports the value of counseling for mothers about the importance of KMC by community health worker [28]. In a systematic review of studies conducted in lower and middle-income countries, understanding of mothers about the benefits of KMC was found to be among top five facilitators to practice KMC at facility and household level [10]. In rural India, most mothers were found to have a good understanding about KMC after counseling by community health workers, and they could demonstrate the process of KMC correctly [29].

Our study participants at the community level were aware that newborns should be protected against the cold, and skin-to-skin care could be a beneficial way to protect these babies at risk but skin-to-skin contact to provide warmth is not the prevailing norm. Researchers in Ghana also found mothers recognizing that low weight babies could result in serious illness or endanger their life, and they believe skin-to-skin contact care could improve their babies’ health and survival [30].

While our study data revealed the feasibility of KMC initiation at two health facilities and continued use at home within these two study districts, we also found concerns and reservations expressed by study participants related to socio-cultural norms, household work responsibilities and privacy. Researchers also identified social and cultural norms as major issues in the adoption of KMC and found that social support can improve the uptake and duration of KMC [28]. Some elders and uneducated people in the community with a conservative mentality, may be reluctant to accept new methods like KMC as they still believe that traditional methods are safe. Even some people who perceive KMC as a beneficial method, are unwilling to practice this due to the fear of criticism by others [31].

Support from family members is very crucial to continue KMC practice, and it is found to be one of the top ranked enablers for KMC implementation, especially in low and middle-income countries [10]. Specifically, support from mother-in-law and sister-in-law is crucial for the recently delivered mother to practice KMC. Given the joint or extended family structure in Pakistan, it is highly likely that other female family members will be able to support the mother to practice KMC. In Brazil, mothers were grateful to have someone help them during kangaroo mother care, such as grandmothers and sisters, who could take care of housework and help with the newborn [32]. A study in Bangladesh reported where mothers felt resistance from family members, mostly from their mother-in-law and sister-in-law to practice KMC [33]. Participants in another study conducted in Bangladesh expressed that getting help from motivated husbands, sisters-in-law, mothers, and mothers-in-laws in holding the neonates skin-to-skin or doing house hold work would facilitate KMC uptake at community [31].

Husbands, specifically in Pakistan, and in other countries with similar sociocultural norms within the region, are usually not the caregivers of the newborns and less likely to have any active role in practicing KMC. Although, husbands showed willingness to practice KMC for the health and survival of their newborns, but it seems they may have little time to practice given their responsibilities of working outside home or they also may suffer challenges of prevailing customs and socio-cultural norm. Due to societal norms, in some places like Zimbabwe, South Africa, and South Asia, fathers feel uncomfortable to take care of their babies or perform KMC, and they see it as only mother’s responsibility [15, 34–36].

The mother’s obligation to perform household chores is one critical barrier to KMC practice [10, 31, 35] and many mothers found KMC as a burden due to responsibilities at home or work [28] which is quite similar to our study findings. In Maharashtra, India, most of the mothers living in a nuclear family could not continue KMC due to their responsibility to perform various household and agricultural work [29] and similar situation was found in Bangladesh too [31].

While our study is one of the very few studies conducted in Pakistan on KMC, specifically no previous exploratory study is reported in the literature exploring barriers and enablers for introducing KMC in health facilities and continuing KMC practice at household in Pakistan. However, there are few limitations in our study. For organizing the FGD sessions, the sampling strategy included only RDWs having an alive baby and thus the RDWs whose baby died before the FGDs were excluded. These excluded RDWs could be contributors of important other information which we had missed. The interviewers were university graduates and that may have influenced study participants to provide socially desirable responses. In an effort to limit such risk, data collectors were trained to take an informal approach to conduct IDIs and FGDs and to collect data objectively (rather than introducing subjective bias). The other limitation of the study may include collecting only qualitative data and skipping quantitative data (either primary data from RDW about their previous and current delivery, age, parity, family home settings, or secondary data from facilities) those could offer additional strength through triangulation of qualitative and quantitative data.

### Recommendation

Based on our study results, and lessons learned from other previous studies reported in literature, to enhance initiating KMC and continuing practice of KMC at health facility in Pakistan, we recommended the following:

- Training of all facility level maternal and newborn care providers on KMC is essential before introduction of facility level KMC in Pakistan. Adapting KMC as additional topic in preservice and in-service training curricula could be one useful aspect in this regard.
- Health facilities need to be fully functional with required equipment, logistics and supplies before initiating and practicing KMC, in addition to performing routine newborn care.
- A behavioral change communication and social mobilization strategy will help creating support for initiation and continued practice of KMC to address the enablers and barriers to individual, household and community level acceptance and practice of KMC in Pakistan. Such a strategy could include a variety of approaches such as interpersonal communications, household visits, identification and use of both male and female community champions, and draw on existing peer groups or local structures. Given the gendered roles of household work and newborn care, enhancing male involvement in KMC practice directly or through sharing household duties to facilitate continued practice by female family members will further support continued practice at household level.
- Additionally, during the introduction of KMC at facilities, specific messages on barriers and enablers need to be focused and customized to targeted audience (e.g., TBA, key influencers, elderly female family members of pregnant women, husbands).
- As a recognized and respected provider of community level health care, the LHWs need to be trained, motivated and encouraged to invest time for ensuring proper implementation of KMC at household level. There is a critical need of creating link between community and health facilities through LHWs for better implementation of KMC. The LHWs covering different village may need transport support to conduct such KMC training and counseling sessions as well as to facilitate mothers to practice KMC.
- Design / development of a culturally appropriate KMC binder and/or KMC friendly dress that meets to local needs for privacy and modesty of mothers and other family members providing KMC. A suitable male KMC binder may also be worth considering. Innovative models of community financing and manufacture, and recycling of KMC binders could be explored to support sustainability and cost-effectiveness.

## Conclusion

Our study results revealed feasibility to initiate KMC practice at health facility and to continue practicing at home after returning from facility in rural communities in Pakistan. Ensuring facility readiness to initiate KMC, improving capacity of health providers both at facility and community levels, coupled with focusing on community mobilization strategy, targeting specific audiences, may help policy makers and program planners to initiate KMC at health facility and keep KMC practice continued at household level in similar settings. Further research could help gathering more insights on these issues.
